# The Air an Anæsthetic

**Published:** 1876-11

**Authors:** W. G. A. Bonwill


### THE AIR AN ANÆSTHETIC.

BY DR. W. G. A. BONWILL.

*Read before the Northern Medical Association, Pa.*

Gentlemen:—By your kind invitation, I am here to-night to give you some information upon a subject which has engaged my attention for many years, and especially so for the last six months. Anæsthesia appeals to the finest feelings of our nature, and I would consider my life had been a blank had I not somewhat investigated it. It has been a hobby with me ever since I began the practice of my profession.

Much of those twenty-one years has been spent in plans calculated to obtund sensibility, without resorting to ether, chloroform, or nitrous oxide gas. To say that I have succeeded at last to my heart's content, would not be true; for I admit of no ultimate to human progress. That I have realized much that is calculated to gratify me for my years of trial, I can not deny. If I can be sure of anything, it is that we have an agent, hitherto overlooked, which is simple, cheap, effective and safe, and it is nothing more nor less than *atmospheric air*, which is capable of sustaining life, or holding it in suspension for a time. The air is to a certain extent, an anæsthetic, which can be used in a large proportion of cases of minor surgery, or as an obtunder of pain in many affections of the body.

Since your invitation, my time has been so occupied, I do not know that I can do more than repeat what I said in my first article before the Franklin Institute, in November last. To this, I will give you my daily experience since, without offering any theory as to its modus operandi, leaving that for the future. I have spoken of it to several of my medical friends, who have been testing it in various ways upon themselves, and upon others. It affords me pleasure to have you know all about it, and if my feeble efforts can be of any value, you are entitled and welcome to them. It is to be hoped you will receive it without prejudice, and investigate and extend the observations.

Well, having observed the good effects of the involuntary efforts of one that is in pain, from the rapid inflating of the lungs, I was led to adopt it in my dental practice, and for at least sixteen years have used it in such manner as I shall show, and with such success that I have been led to carry it a step further. Within the past six months my mind has been specially dwelling upon this subject. I reason thus: If the forcible and occasional inhalation of air could produce the phenomena which I had noticed so long in obtunding sensitive dentine, why may not the rapid voluntary inhalation of it for half a minute or more produce a more decided and permanent effect? If, while the heart is pulsating seventy to the minute, I take in by forced inhalation four or five times the quantity of the mixture, oxygen and nitrogen, what effect will be produced? I said at once that anæsthesia to a certain extent must result. And upon this I at once gave it a trial, which convinced me that there was some change that had come over me. I did so again and again, with similar results. Scarcely a day has passed but a trial in some way has been given it. So paradoxical is it that I have doubted my own senses at each successful application; but I have seen it so often, and had the trials of others related to me, that I am compelled to believe there is an atom of truth in my assertion.

My wife, who has ever been ready to ridicule all my projects, had to testify as to the etheric effects of air. Repeat it as often as she would, the same effect was produced. She could feel the needle which I was thrusting into her flesh, but no pain, and offered no resistance, She was powerless to raise a hand. Muscular exertion was in abeyance. The peculiar sensation, as from chloroform was present,

An old lady of seventy years, and a negro girl of fifteen, in my employ, are easily influenced, and are so powerless that a pin has no effect in arousing them. Each repetition is the same.

Miss A. had been coming to my office for one year, to have a sensitive tooth filled, and each time she went away without having it done. . I had tried to give her gas, but without avail. Finally, I got her to breath rapidly, and I accomplished, without any trouble to either of us, what had so long baffled me.

Miss A. has since informed me she had had an operation performed upon her foot, of five minutes duration, with no other anæsthetic than air, and was again successful, and she was delighted.

Miss S., aged fifteen, was so frightened and excited that I could not pacify her sufficiently to permit me to proceed with cutting the sensitive tooth-bone. I at last prevailed upon her to try rapid breathing, which she did after a few trials. After this it was no trouble at all to perform any operation upon her teeth. I do not think I could have succeeded had I not tried the air.

Miss H., aged twenty-five years, with spinal affection, was so overcome by the attempt at cutting the sensitive bone of a tooth, that I could scarcely prevail upon her to remain in my office. After she inhaled, which she did very perfectly, she was for five minutes so under my control that I cut the bone with impunity, and so profound was the effect that I several times felt the pulse to see if she was all right. After that I extracted a tooth, which required two instruments, used separately, and again with perfect success.

Master M., aged fourteen, had a very large superior first molar extracted, and no evidence of pain was manifested.

A student of the Jefferson Medical College came to me before the close of the lecture and voluntarily testified to the great success he and several of the class had had, and showed me how far into his hand they ran a pin, and left it there until he was again himself, when he was satisfied that no pain resulted when it was done. He declared his perfect confidence in it.

I have extracted on several occasions, for adults and children, and with unusual success.

In addition to my own experience, I had from their own lips the testimony in its favor of several patients at the Pennsylvania Hospital. There were some failure in cases of boys, who could not be induced to breathe rapidly, and of course no effect. I could repeat many other cases, but let these suffice.

So, gentlemen, after seeing and hearing the evidence of so many, I can not longer deny the power of this simple gas. There is very much that might be said in theorizing upon its peculiar effects upon the circulation, brain, and general system, but it can not be done so early in its history; we must wait and watch its phenomena.

Before closing, it is well to mention one other fact in this connection. It has been noticed that, when it is desirable to produce a very profound effect, where ether or chloroform was hitherto necessary, if the subject is made to breathe air as directed, until its effects are produced, and ether be then administered, its effects are manifested at once, and with the smallest possible quantity. From such a course there is no possible danger, as the quantity is so small, and the blood has been so thoroughly hyper-oxygenated, that no fatal or unpleasant results can ensue. If it had no other place in our list of therapeutical agents, it would well repay the application in saving of ether, as well as avoiding so much nausea and unpleasant sequelæ.

Thanking you for your courtesy, all I have to ask is, do not speak lightly of it until you have given it a fair trial, when you will be satisfied "there is something in the air."

A medical friend has just informed me of a case of midwifery, which for twelve or fifteen hours had been in labor, without much dilatation. Left her for several hours and found but little change. Resorted to rapid respiration, and with fingers upon the os, to test its effect, found in three minutes rapid change, and in seven minutes child was born, to the great surprise of the nurse, who expected, from the progress of the case, she would be up all night. It was a beautiful illustration, admitting of no doubt.—*Med. and Surg. Reporter.*

